# Chronic tic cough in adults: a case report

**DOI:** 10.3389/fmed.2025.1657223

**Published:** 2025-08-12

**Authors:** Wanjing Yang, Zixin Qiu, Duanya Liu, Yajing Meng, Changjian Qiu

**Affiliations:** ^1^Mental Health Center, West China Hospital, Sichuan University, Chengdu, China; ^2^Clinical Medicine College of Southwest Medical University, Luzhou, China

**Keywords:** chronic cough, tic disorder, tic cough, multidisciplinary treatment, psychiatric evaluation

## Abstract

Chronic cough in adults is commonly caused by respiratory disorders (e.g., cough variant asthma, CVA), ear, nose and throat (ENT) disorders (e.g., postnasal drip), digestive disorders (e.g., gastroesophageal reflux disease, GERD), as well as anaphylaxis and allergy. Tic cough is infrequent in adults but warrants consideration in individuals who have excluded these somatic disorders and exhibit inadequate response to diagnostic treatments, especially those with a history of tic disorder or ongoing tic symptoms. In these cases, a multi-disciplinary treatment (MDT) for chronic cough that includes psychiatrists is recommended as the optimal management approach.

## Background

Chronic cough is known as a respiratory symptom that lasts more than 8 weeks (1), is often a burdensome condition which is commonly caused by respiratory disorders such as cough variant asthma (CVA), ear, nose and throat (ENT) disorders, digestive disorders, anaphylaxis and allergy (2–11), and features complex causes and leads to insomnia, anxiety, and other symptoms (12–17). About 40% of adults with chronic cough referred for specialist evaluation have persistent cough despite optimal treatment of conditions associated with chronic cough, known as refractory chronic cough (RCC) (18). RCC is a disorder of the brain and the respiratory system (19), in 2020, recent data from tertiary cough specialist clinics have reported prevalence rates up to 60% (20). Currently gabapentin is the first choice in pharmacologic treatment (21). Even after various tests and treatments, it is still difficult to consider the possibility of tic cough (22). Tic cough is a type of vocal tic in tic disorders (23, 24). Tic cough can be characterized by coughing, throat clearing and so on due to the momentary activation of local muscles in the laryngeal part of the pharynx (12). A case of an adult with a tic cough was found in a chronic cough MDT which is reported below.

## Case description

A 32-year-old male presented with a cough 11 years ago after catching a cold in winter, which manifested as a sudden, explosive, persistent dry cough with occasional thin white sputum. He consulted ENT, respiratory medicine, traditional Chinese medicine (TCM), cough specialist outpatient, and other departments, he had been diagnosed with “rhinitis, asthma, upper airway cough syndrome, intractable cough, chronic cough, chronic nonatrophic gastritis (CNAG), and reflux esophagitis,” and he had been treated with loratadine, budesonide for 2 months, antibiotics (He had no recollection of the names or uses of the antibiotics he had taken. He claimed to have undergone chest CT scans at different hospitals prior to taking the medication and said the results were normal.), gabapentin, omeprazole, mosapride and Chinese medicine, which did not relieve the cough. He has never smoked, works in a healthy environment, and has no history of exposure to dust or heavy metals. He had COVID-19 pneumonia in 2022, and he continued to cough after the pneumonia resolved. In addition, he reported that the cough was more pronounced when he focused on it. Gastroscopy was performed 4 years prior, which suggested superficial gastritis and a duodenal bulbar ulcer (stage A2). After regular treatment with proton pump inhibitors, another gastroscopy in the following year suggested CNAG. Eighteen months ago, the individual consulted different departments in the hospital. Chest high-resolution CT revealed a few small inflammatory nodules in both lungs; the pulmonary function test revealed an FEV1/FVC% of 76.93%, with moderate airflow limitation in the small airways and slightly impaired lung function; the results of the bronchial provocation test (BPT), fractional exhaled nitric oxide (FENO: 14 ppb) and allergen tests were negative; and lymphocyte and total IgE levels were normal according to routine blood tests. Sixteen months ago, he received MDT for chronic cough, which involves pneumology, gastroenterology, ENT, allergology, integrated traditional and western medicine, and psychiatry physicians.

**Figure 1:**
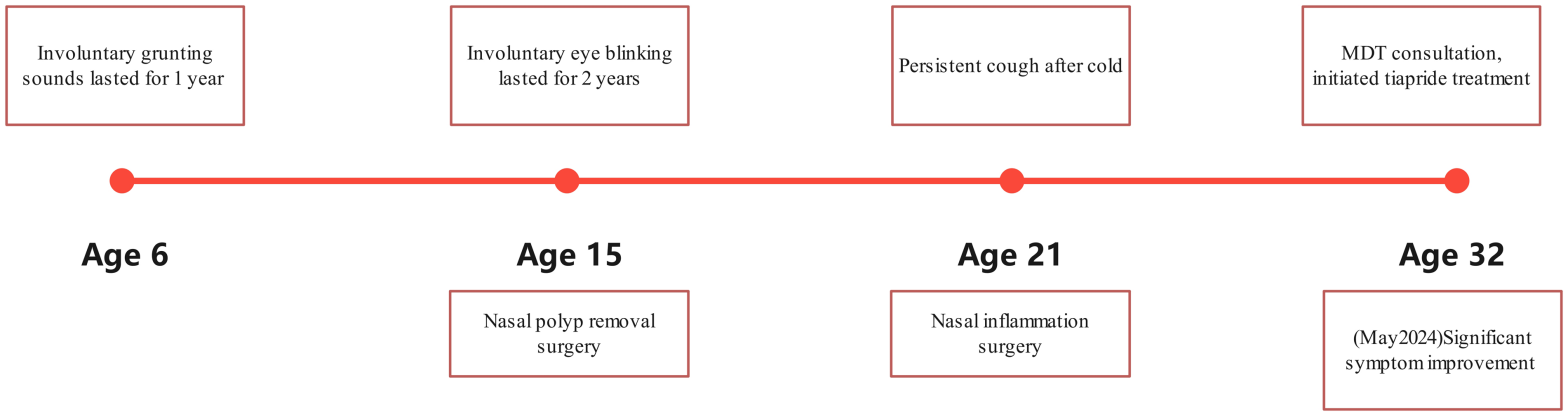
The timeline of the man’s symptom evolution and main intervention.

The MDT ruled out organic disorders and found that he used to have frequent blinking, involuntary head turning, and coughing approximately once a minute for 1–2 s each time, which was similar to “throat clearing.” Psychiatric assessment revealed no depression, anxiety, or insomnia. Past history included involuntary grunting at age 6 (duration: 1 year) and involuntary blinking exacerbated by electronic screens at age 15 (duration: 2 years), previously diagnosed as Tourette syndrome (TS) at a local hospital and resolving post-medication. Notably, the patient’s father had a history of involuntary blinking and unexplained coughing between ages 30–45 years, resolving spontaneously without intervention (Figure 1). During the MDT consultation, the patient was identified and diagnosed with tic disorder by a psychiatrist and recommended the use of medication, and the diagnosis and treatment plan was affirmed and supported by other MDT specialists (respiratory medicine, otolaryngology, gastroenterology). The MDT facilitator then informed the patient of the diagnosis and treatment plan, and the patient expressed agreement and accepted the psychiatrist’s diagnosis and treatment plan.

Tic cough was considered by the MDT, he scored 66 on the Yale Global Tic Severity Scale (YGTSS), the MDT strictly followed the first-line drug guidelines for the treatment of tic disorder, using the most commonly used clinical dopamine receptor antagonists (anti-psychotics), tiapride hydrochloride which was given 0.1 g orally three times daily (tid). Mild symptom improvement at 1 month prompted dose escalation to 0.2 g tid, yielding significant cough reduction. However, bothersome daytime somnolence affecting work led to self-reduction back to 0.1 g tid. At 5 months, the YGTSS score improved to 35 (Table 1), but persistent daytime fatigue prompted self-discontinuation after 7 months, resulting in cough recurrence matching pretreatment severity. Re-initiation of tiapride hydrochloride subsequently improved symptoms again. Leicester cough questionnaire (LCQ) score is used to evaluate the effect of cough on quality of life, the patient’s LCQ score from 65 to 108, the lower the score, the more serious the impact of chronic cough on quality of life is.

**Table 1:** Yale Global Tic Severity Scale (YGTSS).

Date	20-Nov-23		10-May-24	
Classification of tic	Motor	Phonic	Motor	Phonic
Number	3	3	2	2
Frequency	1	5	1	2
Intensity	3	4	2	2
Complexity	1	1	1	1
Interference	1	4	1	1
Score of functional impairment	40		20	
Total motor tic score	9		7	
Total phonic tic score	17		8	
Total tic score (TTS)	66		35	

According to the YGTSS severity scale, a total YGTSS score of ≤25 is considered mild, 25< total YGTSS score ≤50 is considered moderate, and a total YGTSS score of >50 is considered severe. Our patient’s condition was reduced from severe to moderate.

## Discussion

Tic is caused by a pathological enhancement of the motor program driven by abnormally elevated dopaminergic neurotransmission (25, 26). Tic disorders are often diagnosed at ages 4–8 years and are most commonly diagnosed at ages 8–12 years. The condition is predominantly observed in males and typically improves progressively during adolescence and early adulthood (27, 28). Motor tics usually precede vocal tics. Simple motor tics in the earliest stages involve the face, head, or neck (27), such as facial twitching, eye blinking, frowning, head shaking, neck twisting and shoulder shrug. Vocal tics are characterized by sudden, involuntary vocalizations, such as imitating coughing, sneezing, or throat clearing, as well as grunts and wheezes (24, 29). Cough-like symptoms caused by vocal tics are clinically referred to as tic coughs (22). Maria et al. reported that individuals with tic cough accounted for approximately 6% of all children referred for prolonged or recurrent cough (30), and classical antipsychotics are effective against this symptom (31). In China, children with tic-associated coughs, characterized primarily by throat-clearing sounds, are commonly misdiagnosed with respiratory conditions such as postnasal drip syndrome (PNDS) and CVA (32–37). The neuropathological mechanism of tic cough is currently unknown, some of the literature suggests that Tourette’s syndrome is associated with chronic cough, manifested by tic and abnormal corpus callosum (38, 39), further research is required in the future.

Tic disorder symptoms are characterized by recurrence and variability. Consistent with prior research, tic symptoms are highly variable and fluctuate over time (27), with symptom frequency typically decreasing by late adolescence or young adulthood (40–42). However, tics may still recur in adulthood. Sara M. et al. reported 16 individuals with a childhood diagnosis of TS who reemerged with tics in adulthood, with an average latency of 16 years (40). Klawans et al. reported four individuals who presented with a variety of motor and vocal tics before the age of 9 years, with tics resolving completely by the age of 20 years but recurring after the age of 60 years (28). The onset of tic disorders, which are often first observed in children and adolescents, has also been reported in adulthood. However, first episodes in adulthood are much less common than relapses (43–45). Chouinard S et al. conducted a study on 411 individuals with tic disorders and reported that 22 of them first presented with tic disorders after the age of 21 years. A careful review of their medical history further confirmed that 9 had a history of transient tic disorder in childhood, whereas the other 13 had a new onset of tic disorders in adulthood (41). However, few reports on tic cough in adults exist. Esther et al. reported a 27-year-old male with chronic cough that was not treated for 1 year and was successfully treated with low-dose haloperidol after psychiatric consultation (46).

The 2015 CHEST Expert Guidelines recommend replacing the diagnostic terms “habit cough” and “somatic cough” with “tic cough” and “psychogenic cough,” respectively. The term “somatic cough” should be replaced by “psychogenic cough” (22). Tic cough rarely occurs in adulthood. When standard investigations reveal no abnormality and targeted therapies prove ineffective in adults with chronic cough, tic disorders should be considered, particularly in those with a history of tics or concurrent tic symptoms. A chronic cough MDT team with psychiatrists is the optimal model to achieve this goal (47). The MDT is oriented to patients and supported by multidisciplinary experts, which not only provides patients with the best diagnosis and treatment plan (48–50) but also effectively reduces the waste of medical resources (51).

## Conclusion

The possibility of tic cough should be considered in adults with chronic cough, and MDT involving psychiatrists can make a surprising effective.

## Data Availability

The original contributions presented in the study are included in the article/supplementary material, further inquiries can be directed to the corresponding author.
